# Barriers to timely nutrition support in patients with cancer: A scoping review

**DOI:** 10.1002/ncp.70080

**Published:** 2025-11-29

**Authors:** Francesca Tabacchi, Thomas Mitaras, Vasiliki Iatridi, Jonathan Tammam, Eila Watson, Shelly Coe

**Affiliations:** ^1^ Centre for Nutrition and Health, Faculty of Health & Life Science, Oxford Brookes University Oxford UK; ^2^ Department of Dietetics and Therapies Churchill Hospital, Oxford University Hospitals NHS Foundation Trust Oxford UK; ^3^ The Oxford Institute of Nursing, Midwifery and Allied Health Research Oxford UK

**Keywords:** dietetic referrals, late referrals, malnutrition, nutrition support, oncology, service implementation, service provision

## Abstract

In clinical cancer settings, malnutrition can go undiagnosed and patients often receive nutrition care only after they have lost substantial weight or developed severe side effects. Neglecting to provide nutrition care to a patient in a timely manner can lead to increased difficulties in supporting them and to poorer clinical outcomes. The aim of this review was to identify the barriers to timely nutrition support for patients with cancer before and during medical treatment. PubMed and CINAHL platforms were used to search for relevant published literature in June 2022. The search was updated in January 2025. Advanced search was used using the terms “cancer,” “malnutrition,” “nutritional support,” and their synonyms in combination with “under‐recognition” and associated synonyms. The protocol was prospectively registered on OSF Open Science. A total of 4584 records were identified in the databases, and 41 abstracts were selected for full article screening. A total of 19 articles were included in the review. Evidence from the studies indicates that malnutrition identification and dietetic support are not always implemented in current practice. Identified barriers were grouped into four interconnected macro themes: educational barriers, communication barriers, resource barriers, and sociocultural barriers. This scoping review identifies four barriers to early nutrition support in oncological settings and discusses their implications and how they influence each other. Clinical governance should consider and look to address all barriers when trying to implement dietetic support or design pathways in a timely and efficient manner.

## BACKGROUND

Improving the quality of nutrition care provided to people with cancer requires a better understanding of what, in current practice, is preventing them from receiving timely nutrition screening and care. Current guidelines recommend screening for malnutrition risk in all inpatient and outpatient cancer environments and offering support to all patients with cancer at risk of malnutrition.^1^, ^2^ Nutrition screening happens inconsistently in clinical practice, with malnutrition being broadly underrecognized and untreated and with significant regional variations in practice.^3^, ^4^ Because of this, patients sometimes are not offered timely support and consultation with an expert, such as a dietitian or a nutrition specialist.^5^, ^6^ It is therefore important to understand at a deeper level the barriers that are currently preventing nutrition care from being provided in a timely manner. Neglecting to provide nutrition care to a patient in a timely manner can lead to increased difficulties in supporting them and to poorer clinical outcomes. Healthcare settings are complex environments, and therefore, decisions and referrals can be based on incomplete information and personal experience of single professionals.^7^, ^8^ Although guidelines from The European Society for Clinical Nutrition and Metabolism, National Institute for Health and Care Excellence, and other bodies highlight the importance of early nutrition support, implementation remains highly variable.

The aim of this review was to identify the barriers to timely nutrition support to patients in oncology before and during cancer treatment. This review aimed at addressing the following question: “What are the barriers to timely nutrition support in patients with cancer receiving cancer treatment?”

## METHODS

This scoping review was conducted according to the Preferred Reporting Items for Systematic Reviews and and Meta‐Analyses (PRISMA) guidelines.^9^ The review protocol was registered prospectively on OSF Open Science (https://osf.io/bz7wf/). The protocol was designed according to the best practice guidance in Peters et al.^10^ The research question also directed the development of inclusion and exclusion criteria, as suggested by Pollock et al.^11^ The question incorporated the Population, Concept, and Context elements. In this review, the population included all articles considering people living with cancer of any type. The concept was barriers, obstacles, and issues preventing timely nutrition support, and the context was oncology centers, oncological healthcare settings, and systems.^10^

### Proposing a definition for late and missed referrals

In this review, the term “late” nutrition support was defined as “support received after multiple opportunities for earlier nutrition support were missed by clinical staff.” This could happen if a patient had previous contact with a clinic or center, and opportunities were missed to screen, assess, or refer to appropriate dietetic services before the patient actually received it. When patients were not offered nutrition support at all, despite malnutrition or dietary needs, the term “missed” referral or “missed” nutrition support was used in this review. Missed referrals could occur despite the use of screening tools and could have a variety of causes, as discussed below.

### Eligibility criteria

Because the available literature was limited, inclusion criteria were kept broad. Studies were eligible if they met the following:

- Published in English‐language journals
- Any study design
- Conducted on humans
- Involving adults (>18 years old) with a cancer diagnosis (all cancer types)
- Inpatients or outpatients
- Conducted in hospital and/or clinical settings during or before treatment (not in end‐of‐life care or among post‐treatment survivors)
- Reported barriers, causes, or reasons for lack of early identification of malnutrition
- Reported barriers, causes, or reasons for lack of early nutrition support

Exclusion criteria were investigations that included patients without a cancer diagnosis or reporting percentages of patients not receiving nutrition support but not addressing the causes.

### Search strategy

**Figure 1:**
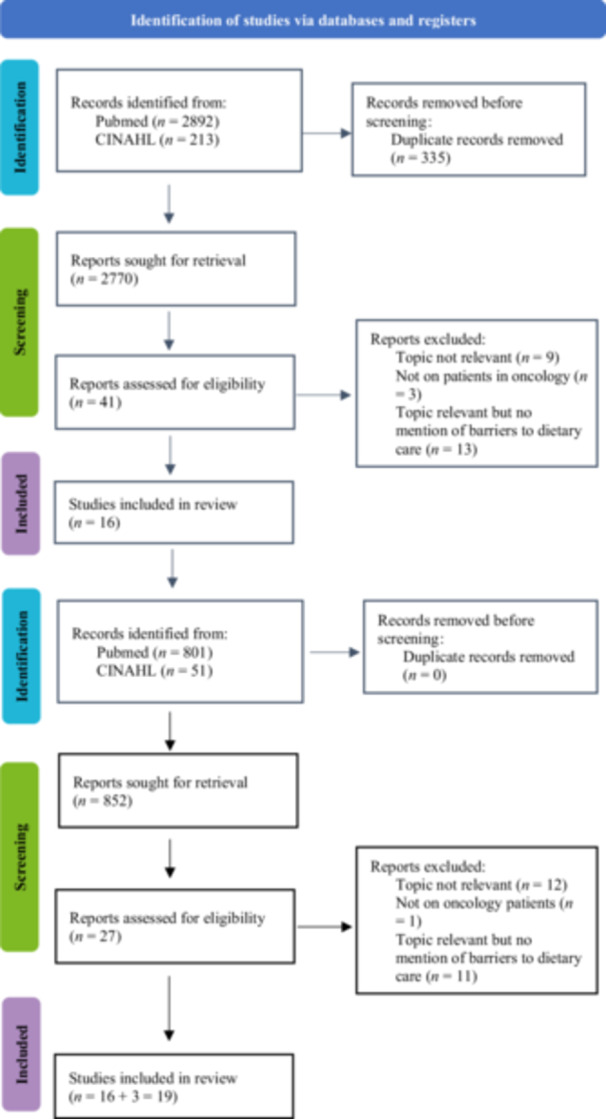
Preferred Reporting Items for Systematic Reviews and Meta‐Analyses flow diagram of the literature search process.

It was clear from preliminary searches that literature on this niche topic was very scarce in comparison to the vast literature on nutrition topics in cancer in general. Furthermore, the lexicon used to treat this topic was variable in articles. A decision was therefore made to keep the search broader and screen more articles to reduce the possibility of missing a potentially relevant study.^12^ Two platforms were used to access electronic databases by two independent reviewers between June and July 2022: PubMed and CINAHL. These platforms were chosen because they all indexed clinical nutrition and nutrition science journals. The search was updated on January 21, 2025, to include any studies published since the last search was performed (illustrated in Figure 1). A time filter was applied to screen articles from the last 10 years because our knowledge of the clinical malnutrition field is continually and rapidly improving. The following search filters were activated: “Humans” and “Aged: 18+ years.” No filter was applied to study design to keep the search as broad as possible.^13^ The search strategy was developed by all coauthors through discussions and preliminary search trials. The university librarian was involved in the design of the search.^14^ An advanced search was performed using the terms “cancer,” “malnutrition,” “nutritional support,” and their synonyms in combination with “under‐recognition” and its synonyms. Supporting Information S2: Appendix S1 and S2 show the detailed search strategy. It was attempted to inquire about the related gray literature by reviewing the reference lists of all full‐text articles included and by contacting some experts and some authors of the papers.^10^

### Study selection

After searching the studies from all databases and eliminating duplicates, the studies were independently screened by two independent researchers. The study selection was conducted in three phases: by title, abstract, and full text of the articles. At each stage, the final decision to include the articles was based on agreement between the authors.^13^ Endnote software online was used to manage the process of the article selection. At the same time, the PRISMA for scoping reviews was applied, as illustrated in Figure 1.^9^

A total of 41 abstracts were selected for full article reading and screening. All 41 studies were read and discussed. From these, 9 were excluded because they did not discuss the topic of nutrition care, 3 were excluded because they addressed a general population without distinguishing between patients with and without cancer, and 13 were excluded because they reported data on lack of nutrition support but without mentioning the causes or reasons behind this. After the updated search of January 2025, consensus was reached that 19 articles should be included in the review.

### Data extraction

Relevant features and results were extracted for each of the included studies.^14^ Country of study, study design, year, aim(s), sample characteristics, and sample cancer diagnosis were extracted, when possible, for all included studies. An Excel spreadsheet was created to extract general data from the articles. Unfortunately, study quality was not evaluated using standard guidelines^15^ because of the diverse study designs, lack of standard malnutrition definition, and inconsistency in defining late or missed referrals (see Table 1).

**Table 1:** Summary of criteria used by the papers to define malnutrition.

Study ID	Criteria used to define malnutrition
Aktas et al. ^16^	The presence of >2 of six criteria: (1) unintentional weight loss (percentage and trajectory); (2) low BMI (<19 kg/m^2^); (3) visible muscle wasting on clinical examination; (4) low nutrient intake (percentage and trajectory); (5) any wound (stages 1–4); and (6) specific abnormal laboratory values (serum prealbumin, serum albumin, or transferrin)
Attar et al. ^17^	HAS malnutrition index (French guidelines)
Baracos et al. ^28^	No definition of malnutrition was provided
Caccialanza et al. ^29^	No definition of malnutrition was provided
Corbaux et al. ^30^	No definition of malnutrition was provided
Deluche et al. ^18^	Malnutrition was defined as weight loss >10% in 6 months, weight loss >5% in 1 month, BMI 18.5 kg/m^2^ or <21 kg/m^2^ if patients
De Waele et al.^19^	>5% weight loss or >2% in individuals with BMI <20 kg/m^2^ (Fearon criteria)
Dijksterhuis et al. ^20^	>5% weight loss or >2% in individuals with BMI <20 kg/m^2^ (Fearon criteria)
Kiss et al. ^36^	No definition of malnutrition was provided
Laing et al.^22^	PG‐SGA
Latenstein et al. ^21^	>5% weight loss or >2% in individuals with BMI <20 kg/m^2^ (Fearon criteria)
Lorton et al.^27^	PG‐SGA and MUST scores
Martin et al.^32^	No definition of malnutrition was provided
Maschke et al.^33^	No definition of malnutrition was provided
Patel et al. ^23^	GLIM criteria and Fearon criteria for cachexia
Raynard et al. ^24^	French national health authorities: moderate malnutrition <70 years old, BMI ≤ 18.5 to 16 kg/m^2^ or SA < 30 to 20 g/L, and ≥70 years old, BMI < 21 to 18 kg/m^2^ or SA < 35 to 30 g/L; and severe malnutrition <70 years old, BMI ≤ 6 kg/m^2^ or SA < 20 g/L, and ≥70 years old, BMI < 18 kg/m^2^ or SA < 30 g/L. Also Fearon criteria for cachexia
Sullivan et al.^25^	No definition of malnutrition was provided
Sun et al. ^26^	Weight loss of >5% over past 6 months, BMI <20 kg/m^2^ and any degree of weight loss >2%, or appendicular skeletal muscle index consistent with sarcopenia and any degree of weight loss >2% (Fearon criteria)
Trujillo et al. ^34^	No definition of malnutrition was provided

Abbreviations: BMI, body mass index; GLIM, Global Leadership Initiative on Malnutrition; HAS, Haute Autorité de Santé; ID, identifier; MUST, Malnutrition Universal Screening Tool; PG‐SGA, patient‐generated Subjective Global Assessment; SA, serum albumin (g/L).

### Data analysis and synthesis

Data on the barriers of nutrition support were extracted and included in the data collection forms in Microsoft Excel. First, thematic analysis was performed to identify common themes in the barriers. Findings from all studies were integrated and collated under four themes. These were then compared and discussed. Once the main themes were identified, another Excel tool was created to identify subthemes and more specific topics. Themes were clarified, tabulated, and discussed with all supervisors with the consultation conforming to the requirements of data synthesis.^13^, ^14^

## RESULTS

Of the 19 included studies, 13 were carried out in Europe. A total of 13 studies involved patients, both inpatients and outpatients, and 6 surveyed views of healthcare professionals. Most studies did not focus on a specific type of cancer. Sample sizes varied by research design, with the range being 50–2375 participants. The characteristics of the studies included are shown in Table 2.

**Table 2:** Overview of key study characteristics.

Study ID	Country	Study design	Type of participants	Sample size, *n* (% women)	Type of cancer
Aktas et al. ^16^	USA	Retrospective observational study	Inpatients	182 (51)	All
Attar et al. ^17^	France	Multicentre cross‐sectional study	Inpatients and outpatients	313 (33)	Gastrointestinal and pancreatic
Baracos et al. ^28^	International	Mixed‐methods study (survey and focus group)	Oncology healthcare professionals	2375	All types
Caccialanza et al. ^29^	Italy	National cross‐sectional study (web‐based survey)	Oncology healthcare professionals and associations of patients with cancer	171 healthcare professionals and 75 patient associations	All types
Corbaux et al. ^30^	France	National cross‐sectional study (expert opinion survey)	Oncology healthcare professionals	206	Lung
Deluche et al. ^18^	France	Cross‐sectional multicentre study	Outpatients	139 (98.6)	Breast
De Waele et al.^19^	Belgium	Retrospective observational study	Inpatients	118	All types
Dijksterhuis et al. ^20^	Netherlands	Multicentre cohort prospective study	Outpatients	406 (23.5)	Gastroesophageal
Kiss et al. ^36^	Australia	National cross‐sectional study (survey)	Oncology healthcare professionals	111	All types
Laing et al.^22^	Australia	Mixed‐methods prospective longitudinal study	Inpatients and outpatients	59 (34)	Gastro‐entero‐pancreatic neuroendocrine tumor
Latenstein et al. ^21^	Netherlands	Prospective multicentre cohort study	Outpatients	202 (47)	Pancreato‐biliary
Lorton et al.^27^	Ireland	Multicentre cross‐sectional study	Inpatients and outpatients	200 (40)	All types
Martin et al.^32^	Europe	Qualitative study	Oncology healthcare professionals	50	Head and neck and esophageal
Maschke et al.^33^	Germany	National cross‐sectional study (online survey)	Outpatients	1335 (76.6)	All types
Patel et al. ^23^	United Kingdom	Retrospective cohort study	Inpatients	232 (67)	Malignant bowel obstructions
Raynard et al. ^24^	France	Cross‐sectional multicentre study	Outpatients	766 (50.1)	All types
Sullivan et al.^25^	Ireland	National cross‐sectional study (survey)	Outpatients and survivors	1073 (63)	All types
Sun et al. ^26^	China	Observational prospective study	Inpatients	390 (41.5)	All types
Trujillo et al.^34^	USA	National cross‐sectional study (survey)	Oncology dietitians	215	All types

Abbreviation: ID, identifier.

### Quantitative findings

A total of 11 studies included in this review reported the prevalence of missed referrals, which ranged from 10%–76%,^16^, ^17^, ^18^, ^19^, ^20^, ^21^, ^22^, ^23^, ^24^, ^25^, ^26^ and 1 study reported the prevalence of late referrals at 45%.^27^ Although these studies examined different cancer populations, used varying definitions of malnutrition and cachexia, and applied different criteria for nutrition support referrals, making a comprehensive quantitative analysis unfeasible, it could be cautiously concluded that, on average, nearly half of patients may not be receiving timely nutrition support.

### Qualitative findings

**Figure 2:**
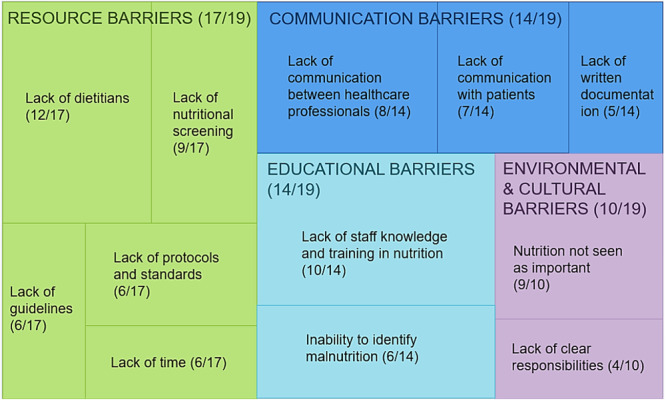
Barriers to early nutrition support in cancer centers. The number of articles reporting each barrier is indicated in brackets. The size of the squares is proportional to the number of studies mentioning these factors as barriers.

The causes of missed or late nutrition support from each article were grouped into four main ones: educational barriers, communication barriers, environmental and cultural barriers, and resource barriers, represented in Figure 2. After thorough discussion among the authors, each barrier was divided into subthemes for significant components (Figure 2). Resource barriers were the most commonly identified causes for missed referrals and were reported in 17 studies (17 of 19), followed by educational (17 of 19), communication (14 of 19) and environmental and cultural (10 of 19) barriers.

### Barrier 1: Resource barriers

Lack of funding, shortages of staffing or time, absence of nutrition pathways or screening, and insufficient literature providing evidence on the importance of nutrition support were all classified as “resources” in this review. All studies but two (17 of 19) reported the scarcity of resources or the resources themselves being suboptimal.^16^, ^17^, ^19^, ^20^, ^21^, ^22^, ^23^, ^25^, ^26^, ^27^, ^28^, ^29^, ^30^, ^31^, ^32^, ^33^, ^34^ Nine studies reported nutrition screening not being performed or being performed rarely or incorrectly,^16^, ^19^, ^20^, ^22^, ^25^, ^27^, ^28^, ^29^, ^30^, ^32^, ^34^ with some authors stressing the lack of a gold standard screening method as well.^20^, ^22^, ^28^, ^32^ Six studies identified the lack of policies and standardized pathways for nutrition support in cancer centers as an important barrier to timely identification of nutrition issues.^20^, ^32^, ^33^, ^34^, ^35^, ^36^ Another four studies indicated that the lack of conclusive literature on the benefits and outcomes of nutrition care was a barrier to the prescription of nutrition support.^28^, ^32^, ^36^ Six studies highlighted the need for more detailed guidelines.^17^, ^22^, ^25^, ^26^, ^28^, ^36^

A total of 12 studies included in this review addressed dietetic staff shortages in cancer centers,^16^, ^19^, ^23^, ^25^, ^27^, ^28^, ^29^, ^30^, ^31^, ^32^, ^33^, ^34^ of which 6 also highlighted the limitations in dietetic services and funding.^23^, ^28^, ^29^, ^32^, ^33^, ^34^ Another five studies identified time pressure of healthcare professionals as a barrier to screening and nutrition care provision.^16^, ^17^, ^27^, ^30^, ^34^

### Barrier 2: Educational barriers

A total of 17 studies reported at least one educational barrier to the early provision of dietetic care in patients with cancer, with most of them identifying the lack of staff knowledge in nutrition as a barrier.^18^, ^20^, ^23^, ^24^, ^26^, ^27^, ^28^, ^30^, ^32^, ^36^ The studies detailed that the lack of knowledge concerns nutrition screening and assessment tools,^27^ the symptoms,^24^ consequences,^28^ or the treatment of malnutrition.^23^, ^26^, ^36^ Four studies mentioned the inadequate training on nutrition support received by healthcare professionals as an educational barrier,^16^, ^28^, ^32^, ^33^ and three studies suggested needing to increase educational campaigns for patients as well.^25^, ^29^, ^32^ Six studies indicated staff inability to identify malnutrition in daily practice or the failure to recognize symptoms impacting nutrition.^16^, ^17^, ^18^, ^20^, ^22^, ^27^ Four studies also reported the lack of staff confidence in recognizing malnutrition or providing support for it,^17^, ^27^, ^28^, ^36^ whereas some highlighted the contrast between staff awareness of the importance of nutrition and their lack of ability to act accordingly in practice. A total of 11 of the studies identified a need to increase staff awareness of nutrition issues and malnutrition identification and treatment.^16^, ^19^, ^20^, ^21^, ^24^, ^27^, ^28^, ^29^, ^30^, ^32^, ^33^

### Barrier 3: Communication barriers

A total of 14 studies addressed the issue of poor communication regarding nutrition issues leading to delays in taking necessary actions.^16^, ^18^, ^19^, ^20^, ^21^, ^24^, ^25^, ^27^, ^28^, ^29^, ^30^, ^32^, ^33^, ^36^ The critical points of communication identified by the studies were poor communication between healthcare professionals and patients, preventing timely identification of nutrition issues; poor interdisciplinary communication among staff; and poor written documentation on patients' oral intake, nutrition status, and symptoms impacting dietary intake. Four studies mentioned patients not being asked about nutrition issues and lifestyle during routine consultations,^18^, ^25^, ^30^, ^36^ and two studies indicated patients underreporting their nutrition issues.^21^, ^27^ Two studies advised clinical staff to assess the nutrition status of patients with cancer at each follow up and urged clinicians to talk more openly about malnutrition to patients.^25^, ^32^ Four studies reported a failure to discuss and communicate across clinicians.^16^, ^19^, ^28^, ^32^ Three studies mention the need to increase the presence of dietitians and/or nutrition topics in multidisciplinary team meetings (MDTs).^19^, ^30^, ^32^ Seven articles advised an improvement in the communication between staff, especially between medical doctors and dietitians, and encouraged frequent collaboration.^16^, ^19^, ^20^, ^24^, ^28^, ^32^, ^33^ One study mentioned staff not knowing how or who to refer patients for nutrition concerns,^35^ and two mentioned referrals being sporadic or delegated.^20^, ^35^ Regarding written communication in medical notes, five articles reported underdocumentation of nutrition status and nutrition information in medical records.^16^, ^19^, ^27^, ^28^, ^30^

### Barrier 4: Environmental, cultural, and occupational barriers

Half of the studies (10 of 19) mentioned at least one environmental element or prejudice that represents a barrier to holistic and comprehensive nutrition care.^16^, ^17^, ^21^, ^23^, ^27^, ^28^, ^30^, ^32^, ^33^, ^36^ Specifically, five of these studies mentioned that oncology staff do not see nutrition care as a priority or as relevant to cancer care.^16^, ^21^, ^28^, ^30^, ^36^ Some of the studies reported that healthcare professionals are traditionally more focused on strictly medical care, and some report nutrition receiving a low level of attention.^16^, ^28^, ^30^, ^33^ Three studies reported nutrition care to not be integrated into standard care or not seen as an essential component of care.^17^, ^21^, ^23^, ^28^, ^32^, ^36^ Four studies mentioned some degree of uncertainty in relation to clinical staff's scope of practice and their responsibilities in nutrition screening and nutrition care.^27^, ^28^, ^32^, ^36^ This uncertainty concerned the responsibility of identifying malnutrition and/or referring patients to dietitians and the staff's involvement in the nutrition care plan. In summary, a concise proposal of actions to address the identified barriers is presented in Table 3.

**Table 3:** Call‐to‐action to resolve identified barriers.

Barrier category	Key barrier identified	Recommended action	Target audience
Resource barriers	Lack of dietitians; lack of nutrition screening; lack of protocols and standards; lack of time and guidelines	Increase funding and staffing for dietitians; implement mandatory nutrition screening at clinical appointments; develop and enforce institutional protocols	Hospital administrators; policymakers
Communication barriers	Poor communication between healthcare professionals; lack of communication with patients; lack of documentation	Ensure dietitians are included in MD teams; integrate structured communication pathways; use standardized nutrition documentation tools	Healthcare teams; clinical managers
Educational barriers	Limited staff knowledge and training; inability to identify malnutrition	Incorporate nutrition education into medical and nursing curricula; provide regular in‐service training on malnutrition screening and management	Medical schools; professional associations; hospital education departments
Environmental and cultural barriers	Nutrition not prioritized; unclear responsibilities	Promote awareness campaigns on the importance of nutrition in cancer outcomes; define and assign clear responsibilities within care teams for nutrition screening and intervention	Health authorities; clinical leadership; oncology teams

## DISCUSSION

Although no single leading cause hindering timely dietary interventions for patients with cancer was revealed, some key categories of barriers that are crucial to patients' dietetic care were suggested and explored. Of the different barriers affecting timely nutrition care, lack of staff education and lack of resources seemed to have the greatest impact on clinical practice because they appeared more frequently in the studies of this review.

The educational barriers that emerged from this review were not new to the scientific literature. Lack of staff training on nutrition is well documented both for nurses and doctors, who are the key contact figures for cancer treatment.^28^ However, the literature also suggests that oncology staff show a strong interest and willingness to receive more education on nutrition and its practical applications.^37^, ^38^ Regarding patients' education, given the current culture and public campaigns largely focusing on the risks of unhealthy eating patterns and obesity, there emerges a lack of awareness and information regarding undernutrition.^39^, ^40^ This factor can lead to underreporting of nutrition issues and misinterpretation of weight loss during cancer treatment. On the other hand, it can result in patients relying on nonscientific sources of information and restricting their diets or using untested supplements.^41^, ^42^

Because medical treatment is the only current potential cure for cancer, oncology staff are historically more dedicated to medical care rather than providing holistic and integrated care. This model implies a low level of involvement of patients, who traditionally do not express their priorities and preferences and do not participate actively in their care.^7^, ^43^ Because of clinical staff's reliance on the biomedical model of care, some literature reports their reluctance to discuss nutrition with patients. To overcome this barrier, some studies suggest integrating nutrition modules into medical and healthcare degree programs and making nutrition a cross‐cutting topic.^29^, ^44^

Looking at the wider literature and into the details of the communication between healthcare professionals, this dialogue can happen at MDTs, which are often not attended by dietitians, or through medical notes.^5^, ^45^ The lack of discussion of nutrition plans between staff could be caused by the implementation of a biomedical model of care, the lack of nutrition knowledge, and/or the lack of confidence of staff in addressing nutrition issues appropriately and in including nutrition in their treatment plans.^46^ Two of the studies analyzed here report a lack of documentation of anthropometrics, which is confirmed in other recent studies.^47^, ^48^ Most studies included in this review recommended recording body weight trends and other anthropometrics such as body mass index (BMI) in the patients' medical records. Some scholars also recommend using standardized language to document and communicate nutrition information, whereas others propose employing more advanced technology and automation, such as shared electronic medical notes, to better use the current data available.^49^, ^50^

As far as the dietetic services are concerned, the lack of dietetic staff is a shared problem across countries, including European industrialized countries, where healthcare is mostly a national public service.^51^ This lack of specialists can in turn lead to reduced awareness about the importance of nutrition care among other clinical staff and to decreased capacity to provide timely nutrition care.^52^ In regard to dietetic screening and pathways, the issues emerged from this review match the ones highlighted in the wider literature. Despite an abundance of literature validating several screening tools in patient populations with cancer, there is evidence that screening can be performed incorrectly.^53^ Additionally, many screening tools assign higher scores to lower BMIs, making them less sensitive for a population with an increasing percentage of overweight and obese patients, who can still develop malnutrition.^54^ Some literature emphasizes that documenting a 6‐month weight history and/or weighing patients at each appointment can be useful tools, in contrast with commonly used screenings that are not used in outpatient environments.^55^, ^56^ Another barrier highlighted is that nutrition screening and care guidelines do not account for different cancer types, which are known to cause different nutrition issues. These generic guidelines encourage a single center's responsibility and decision to develop specific and appropriate protocols and pathways.^57^, ^58^ The dissemination of nutrition knowledge should become structural and integrated into oncology settings so that individual cancer center's efforts can obtain the best results possible.^59^

The findings of this review also highlight that the barriers reported are interconnected and likely to influence each other. For instance, lack of knowledge on malnutrition may lead to practicing a biomedical model of care, the same way the provision of care according to a biomedical model discourages seeking education on nutrition. Equally, not discussing nutrition with patients discourages them from reporting dietary issues and leads them to either seek information elsewhere, often from unreliable resources, or to normalize or even embrace their weight loss. Likewise, the elimination of one of these barriers could lead to an improvement in other areas. For example, an increase in dietetic staffing can improve their capacity to attend MDTs, which could lead to increased nutrition education for other staff and ultimately benefit patients' care. Compulsory documentation of nutrition data or information could lead to broader awareness among staff and more effective communication between staff and patients. Nutrition support pathways indicating specific roles and responsibilities of different clinical staff could increase involvement of staff in nutrition care and enable a shift in culture of care. In turn, more knowledgeable and skilled staff will be more aware of nutrition issues and able to monitor patients carefully, eliminating delays in care. Similarly, when patient education also involves families and caregivers, they can be key in helping monitor and report nutrition issues to staff. To try creating a virtuous circle, resources should be invested in both making nutrition information more available for patients and, at the same time, offer education and training for staff. In turn, more knowledgeable and skilled staff will be more aware of nutrition issues and will be able to monitor patients carefully and avoid delays in care. Likewise, education given to patients should also involve families and caregivers. They will be key in helping monitor and reporting nutrition issues to staff as well. The dissemination of nutrition knowledge should become structural and integrated into oncology settings so that single cancer centers can provide more standardized care and share resources.

In line with these findings and to address the need for more concrete guidance raised by clinicians, several practical steps can be proposed for cancer centers:

- Embed nutrition into routine practice: Make screening and assessment standard, with mandatory documentation.
- Strengthen multidisciplinary teamwork: Ensure dietitians are systematically included in MDTs and treatment planning.
- Enhance education and training: Provide targeted nutrition education for oncologists, nurses, and allied health professionals.
- Promote cultural change: Raise awareness of the role of nutrition in cancer outcomes through campaigns and leadership support.
- Improve resources and infrastructure: Allocate time for dietetic consultations, invest in information technology systems, and collaborate with advocacy groups.

These practical, center‐level steps complement the broader system‐level recommendations outlined in Table 3, which summarizes proposed actions by barrier category and target audience.

### Strengths and limitations

Several studies identified barriers to timely nutrition support in cancer; however, they only focused on some aspects or just a few barriers and did not present a comprehensive and complete mapping of them. Although it is possible that certain barriers may be more prevalent in specific cancer populations, this review was unable to distinguish them, likely because of the limited number of studies included and their mixed populations. Nonetheless, barriers observed in cancer inpatient and outpatient populations seem to overlap. This correspondence could be explained by the fact that patients with cancer tend to have a high rate of admissions, blurring the distinction between inpatient and outpatient populations. Moreover, only two studies addressed palliative patients specifically, so once again it was not possible to explore potential differences in barriers between curative and palliative environments. Finally, comparisons of barriers in different countries or across different cancer sites were not possible within the scope of this review.

This review aimed at identifying all publications available within the topic; however, it is possible that some relevant studies were not included. To reduce this risk, the search terms were kept as broad as possible, and reference lists were also checked. Furthermore, it was not possible to quantify the impact of each barrier on clinical practice because populations, countries, and study designs were very different. A final limitation was the definition of malnutrition because most studies used different criteria to define malnutrition and risk of malnutrition.

## CONCLUSIONS

This scoping review was the first to provide a comprehensive mapping of barriers to timely nutrition support in patients with cancer in clinical practice. The prevalence of missed and late dietetic referrals reported is high and attributed to various interconnected factors including lack of education, lack of communication, sociocultural barriers, and lack of resources. Anywhere cancer care is provided is likely to have a different make up of clinical experts and care protocols, and consequently, a unique combination of the different barriers identified here. To effectively eliminate the risk of missed and late dietetic referrals, it is therefore critical to consider all the different aspects that could apply to a specific environment, tackling various barriers using appropriate strategies. Finally, because nutrition support is not usually used as a sole clinical intervention, pathways designed by research bodies or single policies should involve all stakeholders contributing to patient care to keep a prioritized focus on malnutrition prevention and holistic care.

## AUTHOR CONTRIBUTIONS

Francesca Tabacchi, Shelly Coe, Jonathan Tammam, Vasiliki Iatridi, Eila Watson designed the review protocol and search strategy. Francesca Tabacchi led the conduct and writing of the review. Thomas Mitaras contributed to study screening and identification. Francesca Tabacchi completed data extraction. Shelly Coe, Vasiliki Iatridi, Eila Watson, and Jonathan Tammam reviewed drafts of the review and approved the final submission.

## CONFLICT OF INTEREST STATEMENT

None declared.

## Supporting information

Supplementary file.

Supplementary file with Appendices.
