# Supplementary material for: Barriers to timely nutrition support in patients with cancer: A scoping review
Source: Nutr Clin Pract. 2025 Nov 29;41(2):486–97. doi: 10.1002/ncp.70080 (PMC12982639; doi:10.1002/ncp.70080)
Supplement: Supplementary file 2 — Supplementary file with Appendices. [file NCP-41-486-s002.docx]

# **Barriers to timely nutrition support in cancer patients: a scoping review**

***Appendices***

Appendix 1: Table of synonyms used for the word search

| #1 | #2 | #3 | #4 |
| --- | --- | --- | --- |
| malnutrition | cancer* | underrecognition | nutritional support |
| malnouris* | malign* | unidentif* | nutritional intervention |
| sarcopeni* | tumor* | failure | dietetic support |
| cachexi* | tumour* | identif* | dietetic intervention |
| cachec* | oncolog* | untreated | dietetic service |
| "weight loss" | neoplasm* | recognition | nutritional care |
| anorex* | haematolog* | underestimat* | nutritional advice |
| wasting | hematolog* | inadequate | dietetic consultat* |
| undernutrition | carcinoma* | misdiagnos* | dietitian consultat* |
| undernourish* | chemotherap* | missed diagnosis | nutritional consultat* |
|  | radiotherap* | late diagnosis | nutrition consultat* |
|  | cancer n2 patients | delayed diagnosis | dietitian referral* |
|  |  | late referral* | dietetic referral* |
|  |  | delayed referral* |  |
|  |  | missed referral* |  |

Appendix 2: Literature Search Strategy

("malnutrition"[MeSH Terms] OR "malnutrition"[All Fields] OR "malnutrition s"[All Fields] OR "malnutritional"[All Fields] OR "malnutritions"[All Fields] OR "malnouris*"[All Fields] OR "sarcopeni*"[All Fields] OR "cachexi*"[All Fields] OR "cachec*"[All Fields] OR ("weight loss"[MeSH Terms] OR ("weight"[All Fields] AND "loss"[All Fields]) OR "weight loss"[All Fields]) OR "anorex*"[All Fields] OR ("cachexia"[MeSH Terms] OR "cachexia"[All Fields] OR "wasting"[All Fields]) OR ("malnutrition"[MeSH Terms] OR "malnutrition"[All Fields] OR "undernutrition"[All Fields] OR "undernutritional"[All Fields]) OR "undernourish*"[All Fields]) AND ("cancer*"[All Fields] OR "malign*"[All Fields] OR "tumor*"[All Fields] OR "tumour*"[All Fields] OR "oncolog*"[All Fields] OR "neoplasm*"[All Fields] OR "haematolog*"[All Fields] OR "hematolog*"[All Fields] OR "carcinoma*"[All Fields] OR "chemotherap*"[All Fields] OR "radiotherap*"[All Fields]) AND ("underrecognition"[All Fields] OR "unidentif*"[All Fields] OR ("failure"[All Fields] OR "failures"[All Fields]) OR "identif*"[All Fields] OR "untreated"[All Fields] OR ("recognition, psychology"[MeSH Terms] OR ("recognition"[All Fields]) OR "psychology recognition"[All Fields] OR "recognition"[All Fields] OR "recognitions"[All Fields]) OR "underestimat*"[All Fields] OR ("inadequate"[All Fields] OR "inadequately"[All Fields] OR "inadequates"[All Fields]) OR "misdiagnos*"[All Fields] OR ("missed diagnosis"[MeSH Terms] OR ("missed"[All Fields] AND "diagnosis"[All Fields]) OR "missed diagnosis"[All Fields]) OR ("delayed diagnosis"[MeSH Terms] OR ("delayed"[All Fields] AND "diagnosis"[All Fields]) OR "delayed diagnosis"[All Fields] OR ("late"[All Fields] AND "diagnosis"[All Fields]) OR "late diagnosis"[All Fields]) OR ("delayed diagnosis"[MeSH Terms] OR ("delayed"[All Fields] AND "diagnosis"[All Fields]) OR "delayed diagnosis"[All Fields])) AND ("nutritional support"[MeSH Terms] OR ("nutritional"[All Fields] AND "support"[All Fields]) OR "nutritional support"[All Fields] OR (("nutrition s"[All Fields] OR "nutritional status"[MeSH Terms] OR ("nutritional"[All Fields] AND "status"[All Fields]) OR "nutritional status"[All Fields] OR "nutrition"[All Fields] OR "nutritional sciences"[MeSH Terms] OR ("nutritional"[All Fields] AND "sciences"[All Fields]) OR "nutritional sciences"[All Fields] OR "nutritional"[All Fields] OR "nutritionals"[All Fields] OR "nutritions"[All Fields] OR "nutritive"[All Fields]) AND ("intervention s"[All Fields] OR "interventions"[All Fields] OR "interventive"[All Fields] OR "methods"[MeSH Terms] OR "methods"[All Fields] OR "intervention"[All Fields] OR "interventional"[All Fields])) OR (("dietetics"[MeSH Terms] OR "dietetics"[All Fields] OR "dietetic"[All Fields]) AND ("support"[All Fields] OR "support s"[All Fields] OR "supported"[All Fields] OR "supporter"[All Fields] OR "supporter s"[All Fields] OR "supporters"[All Fields] OR "supporting"[All Fields] OR "supportive"[All Fields] OR "supportiveness"[All Fields] OR "supports"[All Fields])) OR (("dietetics"[MeSH Terms] OR "dietetics"[All Fields] OR "dietetic"[All Fields]) AND ("intervention s"[All Fields] OR "interventions"[All Fields] OR "interventive"[All Fields] OR "methods"[MeSH Terms] OR "methods"[All Fields] OR "intervention"[All Fields] OR "interventional"[All Fields])) OR (("dietetics"[MeSH Terms] OR "dietetics"[All Fields] OR "dietetic"[All Fields]) AND ("service"[All Fields] OR "service s"[All Fields] OR "serviced"[All Fields] OR "services"[All Fields] OR "services s"[All Fields] OR "servicing"[All Fields])) OR ("nutritional support"[MeSH Terms] OR ("nutritional"[All Fields] AND "support"[All Fields]) OR "nutritional support"[All Fields] OR ("nutritional"[All Fields] AND "care"[All Fields]) OR "nutritional care"[All Fields]) OR (("nutrition s"[All Fields] OR "nutritional status"[MeSH Terms] OR ("nutritional"[All Fields] AND "status"[All Fields]) OR "nutritional status"[All Fields] OR "nutrition"[All Fields] OR "nutritional sciences"[MeSH Terms] OR ("nutritional"[All Fields] AND "sciences"[All Fields]) OR "nutritional sciences"[All Fields] OR "nutritional"[All Fields] OR "nutritionals"[All Fields] OR "nutritions"[All Fields] OR "nutritive"[All Fields]) AND ("advice"[All Fields] OR "advices"[All Fields])) OR (("dietetics"[MeSH Terms] OR "dietetics"[All Fields] OR "dietetic"[All Fields]) AND "consultat*"[All Fields]) OR (("dietitian s"[All Fields] OR "nutritionists"[MeSH Terms] OR "nutritionists"[All Fields] OR "dietitian"[All Fields] OR "dietitians"[All Fields]) AND "consultat*"[All Fields]) OR (("nutrition s"[All Fields] OR "nutritional status"[MeSH Terms] OR ("nutritional"[All Fields] AND "status"[All Fields]) OR "nutritional status"[All Fields] OR "nutrition"[All Fields] OR "nutritional sciences"[MeSH Terms] OR ("nutritional"[All Fields] AND "sciences"[All Fields]) OR "nutritional sciences"[All Fields] OR "nutritional"[All Fields] OR "nutritionals"[All Fields] OR "nutritions"[All Fields] OR "nutritive"[All Fields]) AND "consultat*"[All Fields]) OR (("nutrition s"[All Fields] OR "nutritional status"[MeSH Terms] OR ("nutritional"[All Fields] AND "status"[All Fields]) OR "nutritional status"[All Fields] OR "nutrition"[All Fields] OR "nutritional sciences"[MeSH Terms] OR ("nutritional"[All Fields] AND "sciences"[All Fields]) OR "nutritional sciences"[All Fields] OR "nutritional"[All Fields] OR "nutritionals"[All Fields] OR "nutritions"[All Fields] OR "nutritive"[All Fields]) AND "consultat*"[All Fields]) OR ("late"[All Fields] AND "referral*"[All Fields]) OR (("dietitian s"[All Fields] OR "nutritionists"[MeSH Terms] OR "nutritionists"[All Fields] OR "dietitian"[All Fields] OR "dietitians"[All Fields]) AND "referral*"[All Fields]) OR (("dietetics"[MeSH Terms] OR "dietetics"[All Fields] OR "dietetic"[All Fields]) AND "referral*"[All Fields]) OR (("delay"[All Fields] OR "delayed"[All Fields] OR "delaying"[All Fields] OR "delays"[All Fields]) AND "referral*"[All Fields]) OR (("missed"[All Fields] OR "misses"[All Fields] OR "missing"[All Fields] OR "missings"[All Fields]) AND "referral*"[All Fields]))
